# Vitamin D deficiency contributes to the diabetic kidney disease progression via increase ZEB1/ZEB2 expressions

**DOI:** 10.1038/s41387-023-00238-2

**Published:** 2023-07-01

**Authors:** Cláudia Silva Souza, Amanda Lima Deluque, Beatriz Magalhães Oliveira, Ana Lívia Dias Maciel, Cleonice Giovanini, Patrícia Aline Boer, Francisco José Albuquerque de Paula, Roberto Silva Costa, Heloísa Della Colleta Franscecato, Lucas Ferreira de Almeida, Terezila Machado Coimbra

**Affiliations:** 1 Department of Physiology of Ribeirao Preto Medical School, University of Sao Paulo, Ribeirao Preto, Sao Paulo 14049-900 Brazil; 2 Department of Internal Medicine, Faculty of Medical Sciences, State University of Campinas, Campinas, São Paulo 13083-970 Brazil; 3 Department of Internal Medicine of Ribeirao Preto Medical School, University of Sao Paulo, Ribeirao Preto, Sao Paulo 14049-900 Brazil

**Keywords:** Calcium and vitamin D, Risk factors

## Abstract

**Background:**

Diabetic kidney disease (DKD) remains one of the main causes of end-stage renal disease (ESRD) and mortality in diabetic patients worldwide. Vitamin D deficiency (VitDD) is one of the main consequences of different chronic kidney disease (CKD) types and is associated with rapid progression to ESRD. Nevertheless, the mechanisms that lead to this process are poorly understood. This study aimed to characterize a model of diabetic nephropathy progression in VitDD and the epithelial-mesenchymal-transition (EMT) role in these processes.

**Methods:**

*Wistar Hannover* rats received a diet with or without VitD before type 1 diabetes (T1D) induction. After this procedure, the rats were accompanied for 12 and 24 weeks after T1D induction and the renal function, structure, cell transdifferentiating markers and zinc finger e-box binding homeobox 1/2 (ZEB1/ZEB2) contribution to kidney damage were evaluated during the DKD progression.

**Results:**

The results showed an increase in glomerular tuft, mesangial and interstitial relative areas and renal function impairment in VitD-deficient diabetic rats compared to diabetic rats that received a VitD-containing diet. These alterations can be associated with increased expression of EMT markers, ZEB1 gene expression, ZEB2 protein expression and TGF-β1 urinary excretion. Decreased miR-200b expression, an important post-transcriptional regulator of ZEB1 and ZEB2 was also observed.

**Conclusion:**

Our data demonstrated that VitD deficiency contributes to the rapid development and progression of DKD in diabetic rats induced by increase ZEB1/ZEB2 expressions and miR-200b downregulation.

## Introduction

Diabetic kidney disease (DKD) is the most frequent and severe diabetic chronic complication [1]. Renal fibrosis is the endpoint of the progressive lesions in the DKD that can lead to end-stage renal disease (ESRD) [2]. The epithelial-mesenchymal-transition (EMT) is a critical mechanism for the progression of fibrosis in the diabetic kidney [3]. EMT is characterized by the loss of the cell epithelial markers and the acquisition of a mesenchymal phenotype [4]. The transforming growth factor beta (TGF-β) is the main inducer of the EMT process by signaling pathways that are dependent or independent of Smad proteins [5]. During this fibrotic process, the TGF-β leads, among other factors, to the increase of zinc finger e-box binding homeobox 1/2 (ZEB1/2) proteins expressions [5]. ZEB1 and ZEB2 act as transcription co-repressors [6] and can promote cell proliferation, transdifferentiating, migration and invasion [7] which are important events of the EMT. In this sense, studies demonstrated an increase of ZEB1/2 expressions TGF-β1-induced during tubular EMT in vitro [6, 8]. These studies also demonstrated a significant increase in ZEB1/ZEB2 expression in the kidneys of rats with chronic kidney disease (CKD) induced by unilateral ureteral obstruction (UUO) [6, 8]. Sene et al. demonstrated an increase in ZEB2 gene expression in isolated glomeruli in fetal-programmed adult rats with low protein levels independent of increases in TGF-β expression [9].

MicroRNAs (miR) are small endogenous non-coding RNA molecules, with a length of 21–25 nucleotides and act by binding to the gene 3′-UTR region inhibiting translation or inducing the degradation of the mRNA target [10]. MiR-200 family, which includes five members (miR-200a, miR-200b, miR-200c, miR-141 and miR-429) epigenetically regulates both ZEB1 and ZEB2 [11]. The ZEB1 and ZEB2 mRNA have eight and nine sites in the 3′UTRs for the miR-200 family, respectively, that bind to these regions and repress ZEB1/2 transcription and translation, impairing the EMT evolution [6, 12]. It has been demonstrated that downregulation of miR-141, miR-200a, miR-200b, and miR-429 were related to EMT in isolated glomeruli of fetal-programmed adult rats with low protein levels [9]. Additionally, Xiong et al. (2012) showed that miR-200 family all members were downregulated during renal tubular EMT both in vivo in the UUO model and in vitro using NRK-52E cells (rat renal tubular epithelial cells) [8].

VitD deficiency emerged as a risk factor for the development of type one diabetes (T1D) and DKD [13]. The kidney is one of the main sites where VitD changes in its active metabolite, the 1α,25-dihydroxy vitamin D3 [1α,25(H)_2_D_3_] [14]. The main physiological role of 1α,25(H)_2_D_3_ is the maintenance of extracellular levels of calcium (Ca^+^) and phosphorus (P^+^) ions in the body [15]. In addition, it was observed that VitD is important for maintaining the podocyte structure, prevention of EMT and reducing albuminuria [5]. Its deficiency has been widely reported in clinical and experimental studies of CKD associated or not with diabetes mellitus (DM) [15–17]. Even with VitD replacement and current therapies to retard progression to ESRD, mainly based on glycemic and blood pressure control, the incidence of DKD is still increasing. Therefore, this study aimed to evaluate the effect of the VitD deficiency in the progression of DKD and the ZEB1 and ZEB2 contribution to this process in rats with T1D.

## Materials and methods

### Animals, VitD deficiency and diabetes induction

The experiments were approved by the Ethics Committee on the Use of Animals of the Ribeirao Preto Medical School at Sao Paulo University, with protocol number 002/2019. Forty-eight *Wistar Hannover* rats (180–200 g) were housed under controlled environmental conditions (12 h light/12 h dark cycle, 22 ± 2 °C) with food and water *ad libitum*. The rats were divided into two groups with 24 rats/each: one group received a standard diet, that consisted of a ration produced according to the AIN-93G protocol, including 1,000.0 IU/kg of VitD3 (VitD) and another group received a VitD3-free diet (VitDD). The composition of the diets is available in Reeves et al. (1993) [18]. Six weeks after the VitD or VitDD diet introduction, the T1D induction was performed by the administration of a single intraperitoneal injection of streptozotocin (STZ; Sigma Aldrich, USA; 45 mg/kg, diluted in 0.1 M citrate buffer, pH 4.5) after 12 h of fasting. The control groups received a single intraperitoneal injection of diluent. Seventy-two hours after STZ administration, diabetes induction (DI) was confirmed by fasting blood glucose (FBG) evaluation. All rats presented FBG equal to/or greater than 250 mg/dL and were considered diabetic [19–24]. After that, the rats remained for 12 or 24 weeks fed a VitD or VitDD diet. The rats were then subdivided into 4 groups: control fed with a VitD diet (Ctrl VitD; *n* = 10), control fed with a VitDD diet (Ctrl VitDD; *n* = 10), diabetic fed with a VitD diet (DM VitD; *n* = 14), and diabetic fed with a VitDD diet (DM VitDD; *n* = 14). At 12 and 24 weeks after vehicle or STZ injections, the rats were euthanized under anesthesia using xylazine (0.1 ml/100 g) and ketamine (0.05 ml/100 g). All diabetic rats received 1 U/day of NPH insulin (Humunlin^®^, Lilly) to maintain a blood glucose level of around 300–350 mg/dL simulating a decompensated blood glucose that occurs in humans [25].

### Assessments of 25-hydroxyvitamin D, glycemia, parathyroid hormone, Ca^+^, and P^+^ plasma and mean arterial pressure

Serum 25-hydroxyvitamin D [25(OH)D] was evaluated by the direct competitive test based on the chemiluminescence principle (CLIA) (DiaSorin, Liaison^®^, Saluggia, Italy). Plasma glucose level was measured using a blood glucose monitoring system (ACCU-CHEK Active, Roche Diabetes Care GmbH). Parathyroid hormone (PTH) was determined in serum samples by Enzyme-Linked Immunosorbent Assay (ELISA) kit according to the manufacturer’s guidelines (Rat Intact PTH ELISA—Quidel, USA). The Ca^+^ and P^+^ in the plasma and urine were quantified by colorimetric method (Kit Labtest Diagnostica S.A) and the excretion fractions of these ions were calculated. Mean arterial pressure (MAP) measurements were performed every 4 weeks by tail plethysmography (CODA *Non-Invasive Blood Pressure System*, Kent Scientific Corporation, 2010).

### Renal function

The glomerular filtration rate (GFR) was estimated by the creatinine clearance. Creatinine plasma and urine levels were determined using the colorimetric method (Kit Labtest Diagnostica S.A). Urinary albumin excretion (UAE) was evaluated by electroimmunoassay [26].

### Renal structure

Kidney fragments collected at the time of euthanasia were immersed in a methacarn solution (60% methanol, 30% chloroform and 10% acetic acid) for 24 h. After this period, the solution was replaced by 70% alcohol. Kidney tissue was embedded in paraffin and histological sections 4 µm thick were stained with Periodic Acid Methenamine Silver (PAMS) and Masson’s Trichrome (MT). PAMS staining was used to determine glomerular tufts areas and fractional mesangial areas evaluated in 50 glomeruli of the renal tissue biopsy in consecutive microscopic fields (0.267 mm^2^). The relative interstitial area was determined by the quantification of the fibrosis area in 30 consecutive microscopic fields (0.267 mm^2^) in the renal cortex and 20 consecutive microscopic fields in the outer medulla from tissue stained with MT. The results were expressed as a percentage of the area marked for MT corrected for the total area of the field [27]. The analysis corresponding to glomerular tufts, mesangial expansion and fibrosis areas was performed by computerized morphometry using the ImageJ 1.44p program (National Institute of Health, USA). The images were acquired using the Zeiss Axio microscope coupled with an AxioCam MRc (AxioVision Release 4.8.3; Zeiss, Germany).

### Immunohistochemistry studies

Immunohistochemistry studies were performed on fragments of 4-µm-thick paraffinized renal tissue mounted on glass slides coated with Entellan™ (Merck Millipore). Kidney sections were incubated overnight at 4 °C with the following primary antibodies: anti-desmin (1:100, Dako, Denmark), anti-vimentin (1:200, Dako, Denmark) and anti-alpha-smooth muscle actin (α-SMA, 1:200, Dako, Denmark). Secondary antibodies were used according to the primary antibody. The sections of renal tissue were revealed with avidin-biotin-peroxidase complex (Vector Laboratories, USA) and 3,3-diaminobenzidine (DAB) (Sigma, USA). The sections were counterstained with methyl green. The evaluations of the immunoreaction for desmin (50 glomeruli), vimentin (30 cortical fields), and alpha-smooth muscle actin (α-SMA, 30 cortical and 20 outer medulla interstitial fields) were performed by computational morphometry using ImageJ 1.44p program (National Institute of Health, USA) and the results expressed as the percentage of the marked area for each of these antibodies. The results corresponding to the desmin immunostaining area were corrected for the corresponding glomerular tuft area and vimentin and α-SMA for the total area of the cortex and medulla per field. The average values per sample were calculated and expressed as a percentage [27].

### Western blotting studies

Renal tissue homogenate was obtained from a lysis buffer (50 mM Tris–HCl, pH 7.4, 150 mM NaCl, 1% Triton X-100, 0.1% SDS, 1 µg/mL aprotinin, 1 µg/mL leupeptin, 1 mM phenylmethylsulphonyl fluoride, 1 mM sodium orthovanadate, pH 10, 1 mM sodium pyrophosphate, 25 mM sodium fluoride, 0.001 M EDTA, pH 8) containing a cocktail of protease inhibitors (Sigma, USA). Samples were incubated at 4 °C for 15 min and then centrifuged at 2000g and protein concentrations were quantified using the Bradford assay method (Quick Start™ Bradford 1x Dye Reagent—Bio-Rad) [28]. Proteins were separated on a 10% polyacrylamide gel, transferred to a nitrocellulose membrane overnight at 4 °C, and blocked for 60 minutes with 5% skim milk diluted in TBST (Tris, NaCl, EDTA/L, and Tween-20), and incubated with the following primary antibodies: anti-VitD receptor (VDR, 1:500, Santa Cruz Biotechnology, USA), anti-ZEB2 (1:200, Santa Cruz Biotechnology, USA) and anti-Smad2/3 (1:200, Santa Cruz Biotechnology, USA). To assess the equivalence of protein loading and/or transfer, membranes were also incubated with anti-GAPDH polyclonal antibody (1:1000, Cell Signaling, USA). Finally, the membranes were washed and incubated with polyclonal anti-rabbit IgG (1:5000, Dako, Denmark) or anti-mouse monoclonal IgG antibodies (1:5000, Dako, Denmark) conjugated to peroxidase for 1 h at room temperature. Membrane-bound antibodies were detected using the chemiluminescent substrate Supersignal West Pico (Pierce Chemical, Rockford, IL, USA). The intensity of the bands was identified and quantified by densitometry using ImageJ NIH software and the results were presented in arbitrary units.

### ZEB1/2 and miR-200a/b/c gene expression

Gene expression studies were performed as described by Sene et al. [9]. Briefly, RNA total renal tissue was extracted from all groups (*n* = 5/each) using Trizol reagent (Invitrogen, USA), according to the manufacturer’s guidelines, and quantity was determined using Agilent Epoch Microplate Spectrophotometer (BioTek Instruments, USA). Reverse transcription (RT) of ZEB1/2 and miR-200a/b/c was performed using High Capacity RNA-to-cDNA Master Mix (Life Technologies, USA) and TaqMan^®^ microRNA RT kit combined with Stem-loop RT Primers (Life Technologies, USA), according to the instructions specified by the manufacturer, respectively. Real-time quantitative polymerase chain reaction (RT-qPCR) was used for the analysis of ZEB1/2 mRNAs and miR-200a/b/c expressions. The RT-qPCR for ZEB1/2 was carried out with SYBR green^®^ Master Mix (Merck, Germany) and miR-200a/b/c was performed using TaqMan^®^ MicroRNA Assay (Life Technologies, USA). The StepOne™ Real-Time PCR System (Applied Biosystem, USA) was used to perform the RT-qPCR reactions. The differential expression of mRNA for ZEB1/2 and miR-200a/b/c was compared between the DM VitD and DM VitDD groups and their respective control groups. Normalization of ZEB1/2 mRNA expression was performed by GAPDH and for miR-200a/b/c by small nuclear RNA (snRNA) U6 and U87 reference genes. Relative gene expression was evaluated by comparative quantification using StepOne™ v2.1 Software (Applied Biosystem, USA) and the 2^-ΔΔCT^ method. All primer sequences used are available in Sene et al (2013) [9].

### TGF-β1 measurements

The TGF-β1 levels were measured in renal tissue lysate (as described in the section on western blotting studies) and in urine samples from the bladder. This profibrotic cytokine was measured in both biological materials using ELISA kits according to the manufacturer’s guidelines (Promega, USA). Bladder urine was treated with 1 mM phenylmethylsulfonyl fluoride (PMSF, Sigma, USA) after collection and stored at −70 °C until analysis [28].

### Statistical analysis

The results were expressed as mean ± standard error of the mean (SEM) and with medians and percentiles 25 and 75. The data with normal distribution were submitted to the Two-Way Analysis of Variance followed by the Bonferroni multiple comparison test. Data that did not present a normal distribution were subjected to the Kruskal–Wallis non-parametric test followed by Dunn multiple comparisons test. Student’s *t* test was used for intragroup comparisons between different experimental times. The level of significance was set at *P* < 0.05. Statistical analyzes were performed using Graph Pad Prism version 9.0 for Windows (Graph Pad Software, La Jolla, USA).

## Results

### Experimental model characterization

The rats of Ctrl VitDD and DM VitDD groups were deficient in VitD at baseline (Time 0), 12 and 24 weeks after diabetes induction or citrate injection (*P* < 0.0001) (Supplementary Fig. 1A). Fasting blood glucose (FBG) was maintained between 300 and 350 mg/dL in diabetic animals throughout the experimental protocol (*P* < 0.0001) (Supplementary Table 1). There were no statistical differences in serum PTH levels, in Ca^+^ and P^+^ plasma between the experimental groups of 12 weeks and 24 weeks (*P* > 0.05) (Supplementary Fig. 1B–D). Eight weeks after diabetes induction, was observed a gentle increase in MAP in the DM VitDD group when compared to the Ctrl VitDD and DM VitD groups (*P* = 0.04) (Supplementary Table 1). The rats of Ctrl VitDD and DM VitDD presented a significant reduction of VDR protein expression 24 weeks after diabetes induction when compared to Ctrl VitD and DM VitD groups (*P* = 0.0007) (Supplementary Fig. 1E).

### Kidney function

**Fig. 1:**
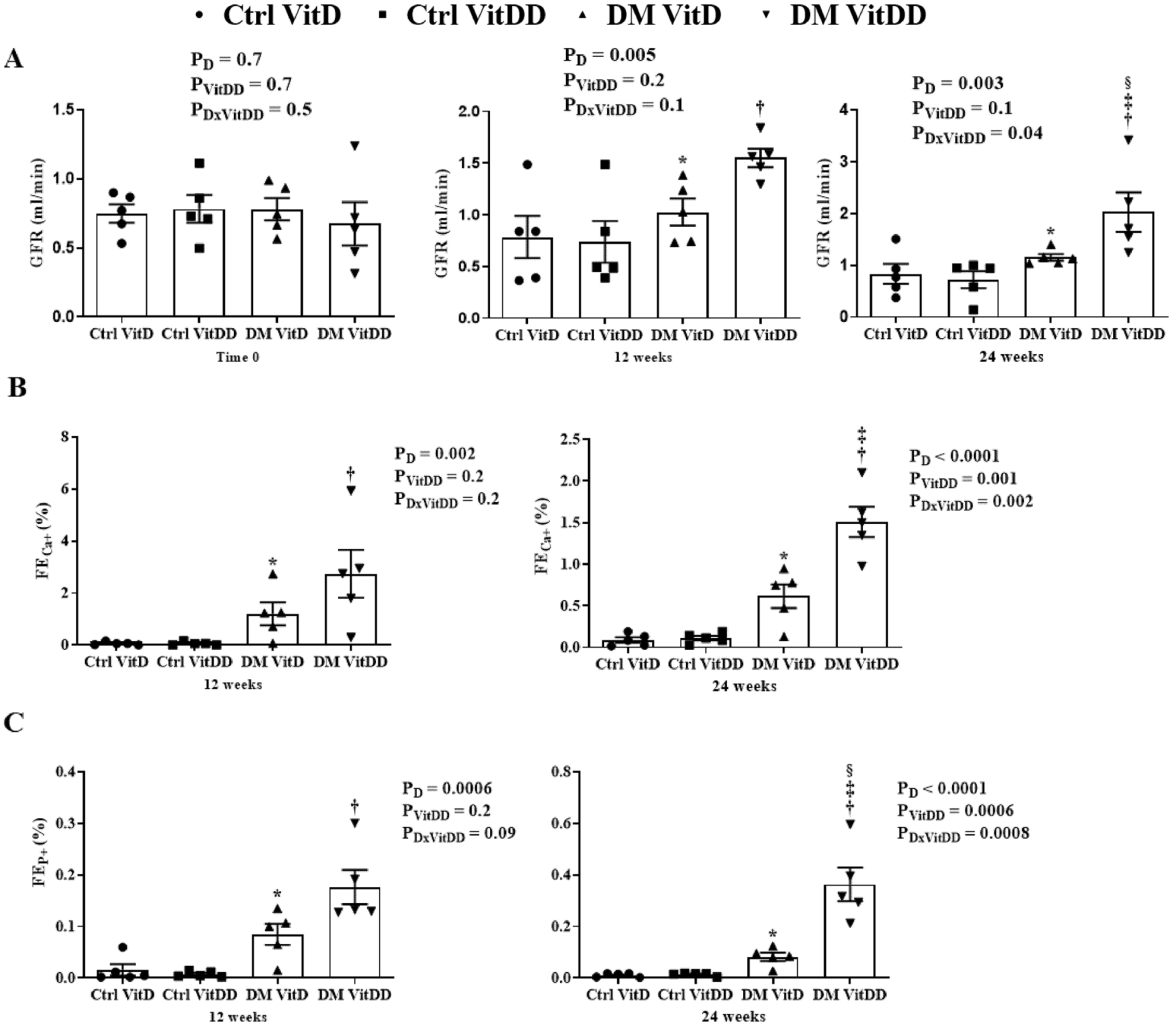
Renal function of control (Ctrl) and diabetic (DM) rats deficient in vitamin D (VitDD) or not (VitD). **A** Glomerular Filtration Rate (GFR). **B** Fractional Excretion Calcium (FECa^+^). **C** Fractional Excretion Phosphorus (FEP^+^). *versus Ctrl VitD, ^†^versus Ctrl VitDD, ^‡^versus DM VitD, ^§^versus DM VitDD of 12 weeks after diabetes induction. *P*_D_ interaction between DM VitD and Ctrl VitD and DM VitDD and Ctrl VitDD; *P*_VitDD_ interaction between Ctrl VitDD and Ctrl VitD and DM VitDD and DM VitD; *P*_DxVitDD_ interaction between DM VitDD and DM VitD.

The rats of DM VitD and DM VitDD groups showed an increase in GFR at 12 weeks after diabetes induction when compared to their respective controls (*P* = 0.005) (Fig. 1A). Twenty-four weeks after the diabetes induction, VitD deficiency contributed to the GFR remained high in the DM VitDD group compared to CtrlVitD and DM VitD groups (interaction between diabetes and VitDD, *P* < 0.04) (Fig. 1 A). A progressive increase in UAE was observed in DM VitD and DM VitDD groups when compared with their respective controls (*P* < 0.05) (Supplementary Table 1). An increase in the fractional excretion calcium (FECa^+^) (*P* = 0.002, *P* = 0.002, respectively) and phosphorus (FEP^+^) (*P* = 0.0006, *P* = 0.0008, respectively) at 12 and 24 weeks after the diabetes induction in the DM VitDD group was evidenced when compared to the Ctrl VitDD and DM VitD groups (Fig. 1B, C). At 24 weeks after the diabetes induction, the increase in the fractional excretion of these ions was directly influenced by VitD deficiency (interaction between diabetes and VitDD, *P* = 0.002 and *P* = 0.0008, respectively) (Fig. 1B, C).

### Kidney structure

**Fig. 2:**
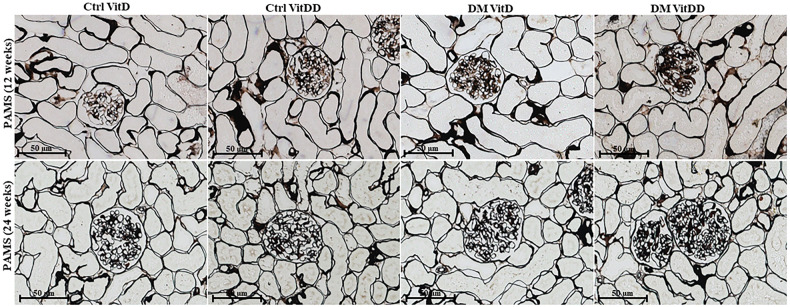
Representative photomicrographs of glomerular histological changes staining with Periodic Acid Methenamine of Silver (PAMS) of control (Ctrl) and diabetic (DM) rats deficient in vitamin D (VitDD) or not (VitD) at 12 and 24 weeks after diabetes induction. Original magnification ×400.

**Fig. 3:**
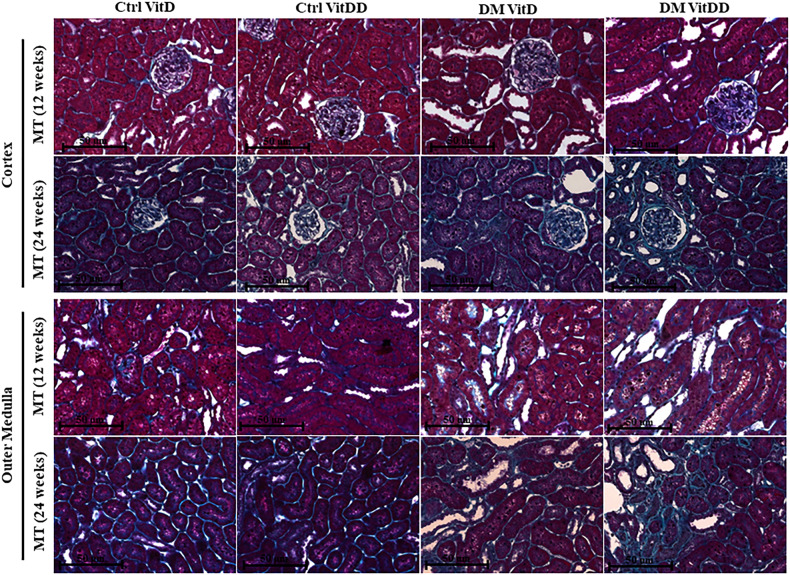
Representative photomicrographs of relative interstitial area staining with Masson’s Trichrome (MT), of control (Ctrl) and diabetic (DM) rats deficient in vitamin D (VitDD) or not (VitD) at 12 and 24 weeks after diabetes induction. Original magnification ×400.

The morphometric data showed an increase in the glomerular tuft area in DM VitD and DM VitDD groups 24 weeks after diabetes induction compared to the respective controls (*P* < 0.0001). Nevertheless, this change was higher in diabetic VitD-deficient rats than in diabetic rats with normal VitD levels (interaction between diabetes and VitDD, *P* < 0.0001) (Table 1, Fig. 2). Vitamin deficiency also led to an increase in fractional mesangial, cortical and medullary relative tubulointerstitial areas in the DM VitDD group when compared to the Ctrl VitDD and DM VitD groups 24 weeks after diabetes induction (interaction between diabetes and VitDD, *P* = 0.0002, *P* = 0.02, *P* < 0.0001, respectively) (Table 1, Figs. 2, 3). No statistical differences in these changes between the experimental groups of 12 weeks were observed (*P* > 0.05) (Table 1, Figs. 2, 3).

**Table 1:** Histology renal data of controls (Ctrl) and diabetic (DM) rats vitamin D deficients (VitDD) or not (VitD).

Variable	GROUP				ANOVA		
	Ctrl VitD	Ctrl VitDD	DM VitD	DM VitDD	*P*_D_	*P*_VitDD_	*P*_DxVitDD_
*Glomerular tuft área (µm*^*2*^*)*							
12 weeks	5097 ± 182	5577 ± 324	5764 ± 109	5965 ± 200	0.05	0.1	0.5
24 weeks	5799 ± 22	5648 ± 98	6550 ± 86^a^	7148 ± 175^b,c^	0.003	0.06	< 0.0001
*Fractional mesangial area*							
12 weeks	0.06 ± 0.06	0.07 ± 0.01	0.07 ± 0.01	0.08 ± 0.03	0.5	0.7	0.5
24 weeks	0.1 ± 0.01	0.1 ± 0.03	0.13 ± 0.01^d^	0.18 ± 0.01^b,c,d^	0.08	0.02	0.0002
*Relative interstitial área (%)*							
*Cortex*							
12 weeks	0.0(0.0;0.6)	0.0(0.0;0.8)	0.4(0.2;0.8)	0.4(0.0;1.2)			
24 weeks	1.2 ± 0.2	1.6 ± 0.7	3.7 ± 1.0^d^	11 ± 2.6^b,c,d^	0.001	0.09	0.02
*Medulla*							
12 weeks	0.0(0.0;0.5)	0.0(0.0;0.8)	0.6(0.1;0.8)	0.6(0.0;1.4)			
24 weeks	3.3 ± 0.4	3.2 ± 0.8	8.6 ± 1.3^d^	14 ± 2.4^b,c,d^	0.06	0.07	< 0.0001

Table 1. Data are presented as mean ± SEM and in median (25th and 75th percentile), *n* = 5/each.

^a^versus Ctrl VitD.

^b^versus Ctrl VitDD.

^c^versus DM VitD.

^d^versus respective 12 weeks group. PD: interaction between DM VitD and Ctrl VitD and DM VitDD and Ctrl VitDD; PVitDD: interaction between Ctrl VitDD and Ctrl VitD and DM VitDD and DM VitD; PDxVitDD: interaction between DM VitDD and DM VitD.

### EMT expression

**Fig. 4:**
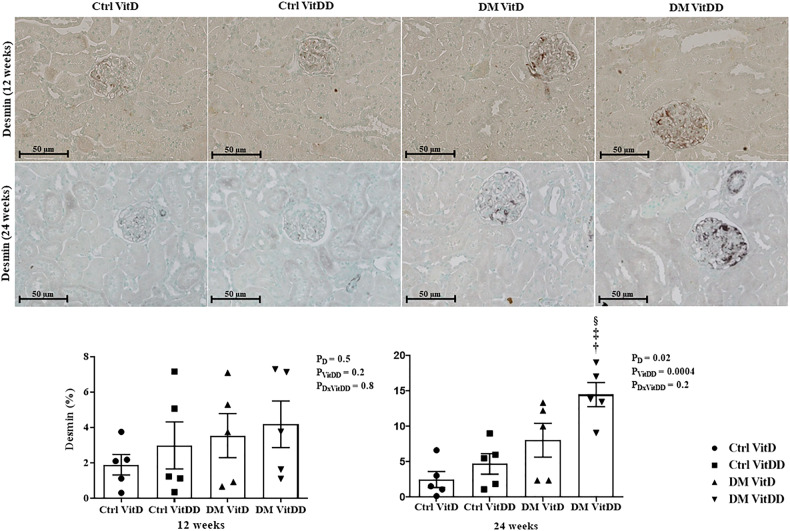
Immunolocalization and quantification for desmin in the glomeruli of controls (Ctrl) and diabetic (DM) rats vitamin D deficient (VitDD) or not (VitD) at 12 and 24 weeks after diabetes induction. ^†^versus Ctrl VitDD, ^‡^versus DM VitD, ^§^versus DM VitDD of 12 weeks after diabetes induction. *P*_D_ interaction between DM VitD and Ctrl VitD and DM VitDD and Ctrl VitDD; *P*_VitDD_ interaction between Ctrl VitDD and Ctrl VitD and DM VitDD and DM VitD; *P*_DxVitDD_ interaction between DM VitDD and DM VitD. Original magnification ×400.

**Fig. 5:**
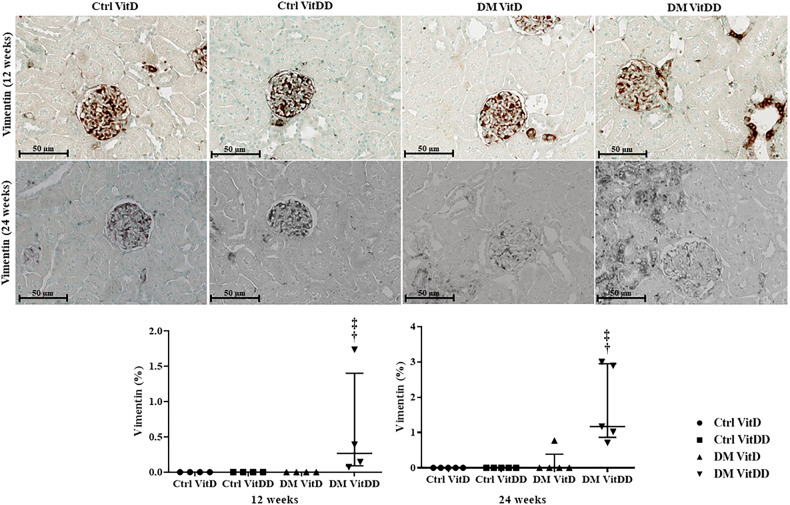
Immunolocalization and quantification for vimentin in the cortex of controls (Ctrl) and diabetic (DM) rats vitamin D deficient (VitDD) or not (VitD) at 12 and 24 weeks after diabetes induction. ^†^versus Ctrl VitDD, ^‡^versus DM VitD. *P*_D_ interaction between DM VitD and Ctrl VitD and DM VitDD and Ctrl VitDD; *P*_VitDD_ interaction between Ctrl VitDD and Ctrl VitD and DM VitDD and DM VitD; *P*_DxVitDD_ interaction between DM VitDD and DM VitD. Original magnification ×400.

**Fig. 6:**
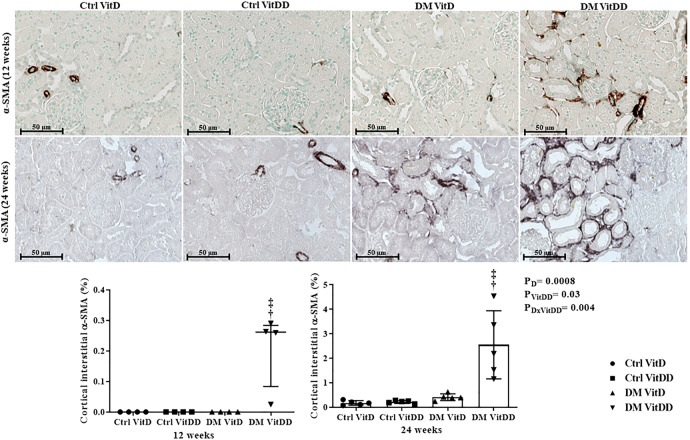
Immunolocalization and quantification for α-SMA in the cortex of controls (Ctrl) and diabetic (DM) rats vitamin D deficient (VitDD) or not (VitD) at 12 and 24 weeks after diabetes induction. ^†^versus Ctrl VitDD, ^‡^versus DM VitD. *P*_D_: interaction between DM VitD and Ctrl VitD and DM VitDD and Ctrl VitDD; *P*_VitDD_ interaction between Ctrl VitDD and Ctrl VitD and DM VitDD and DM VitD; *P*_DxVitDD_ interaction between DM VitDD and DM VitD. Original magnification ×400.

The DM VitDD group showed a significant increase in desmin immunostaining at 24 weeks after diabetes induction (*P* = 0.0004) (Fig. 4) compared to rats of the Ctrl VitDD and DM VitD groups. The same observation was made for vimentin at 12 and 24 weeks after diabetes induction (*P* = 0.002, *P* = 0.001, respectively) (Fig. 5). Increased immunostaining for cortical α-SMA at 12 weeks after diabetes induction was observed when compared to rats of the Ctrl VitDD and DM VitD groups (*P* = 0.002) (Fig. 6). Furthermore, VitD deficiency led to increase in the cortical and outer medulla expression of α-SMA at 24 weeks after diabetes induction when compared to the Ctrl VitDD and DM VitD groups (interaction between diabetes and VitDD, *P* = 0.004, *P* = 0.03, respectively) (Fig. 6, Supplementary Fig. 2).

### ZEB1/2 and miR-200a/b/c gene expression

**Fig. 7:**
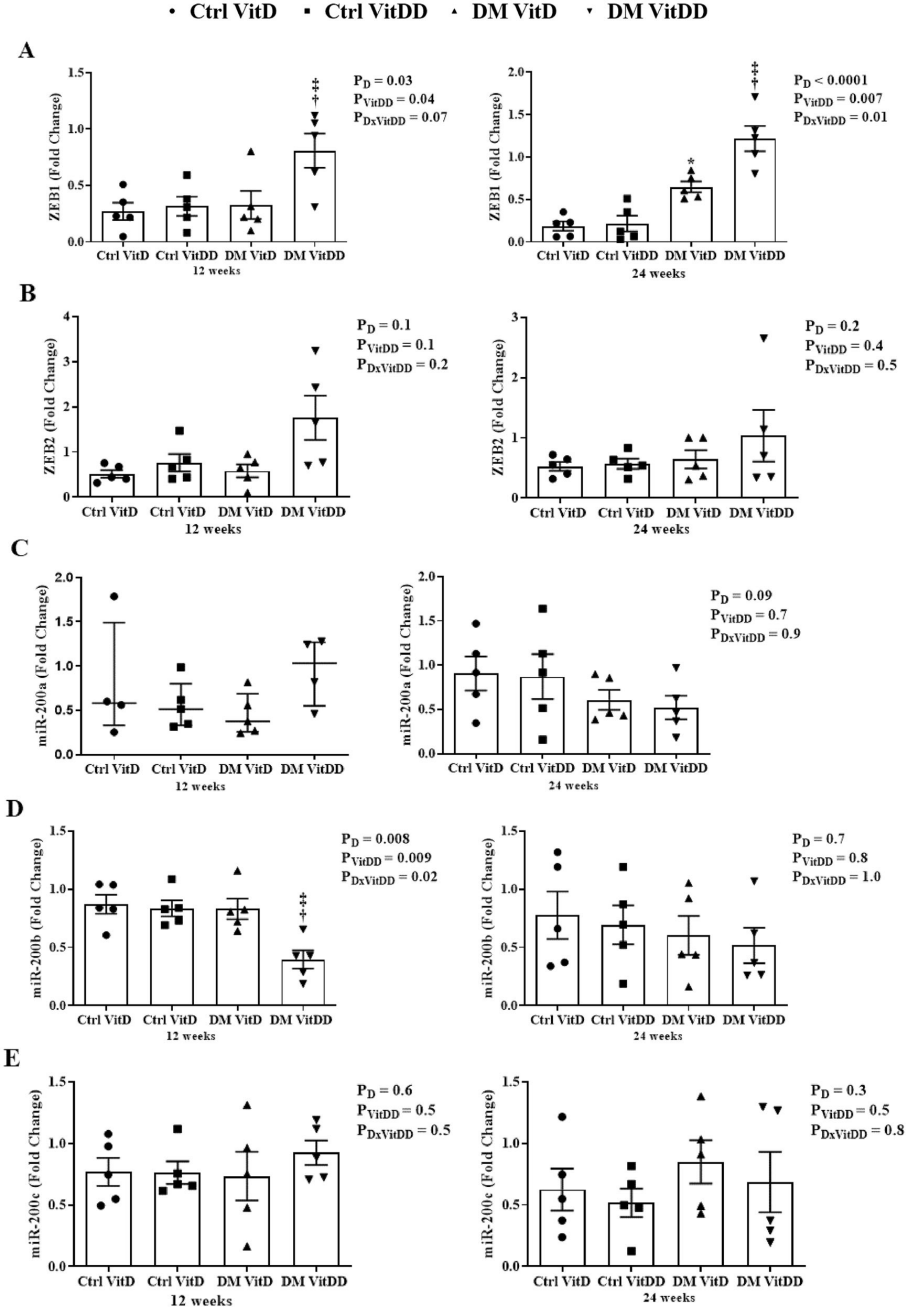
Gene expression of the epithelial-mesenchymal transition process regulators in renal tissue of controls (Ctrl) and diabetic (DM) rats vitamin D deficient (VitDD) or not (VitD) at 12 and 24 weeks after diabetes induction. **A** ZEB1, **B** ZEB2, **C** miR-200a, **D** miR-200b, and **E** miR-200c transcripts levels. ^†^versus Ctrl VitDD, ^‡^versus DM VitD. PD: interaction between DM VitD and Ctrl VitD and DM VitDD and Ctrl VitDD; PVitDD: interaction between Ctrl VitDD and Ctrl VitD and DM VitDD and DM VitD; PDxVitDD: interaction between DM VitDD and DM VitD.

The DM VitDD group presented an increase in ZEB1 gene expression at 12 weeks (*P* = 0.04) after diabetes induction compared to Ctrl VitDD and DM VitD groups (Fig. 7A). At 24 weeks, VitD deficiency favored increased expression of ZEB 1 in the DM VitDD group compared to Ctrl VitDD and DM VitD groups (interaction between diabetes and VitDD, *P* = 0.01) (Fig. 7A). Vitamin D deficiency also led to a reduction in miR-200b gene expression in the DM VitDD group compared to Ctrl VitDD and DM VitD groups at 12 weeks after diabetes induction (interaction between diabetes and VitDD, *P* = 0.02) (Fig. 7D). No statistical difference was observed for ZEB2 mRNA, miR-200a/c between the experimental groups either (Fig. 7B, C, E).

### ZEB2 and Smad2/3 protein expression

**Fig. 8:**
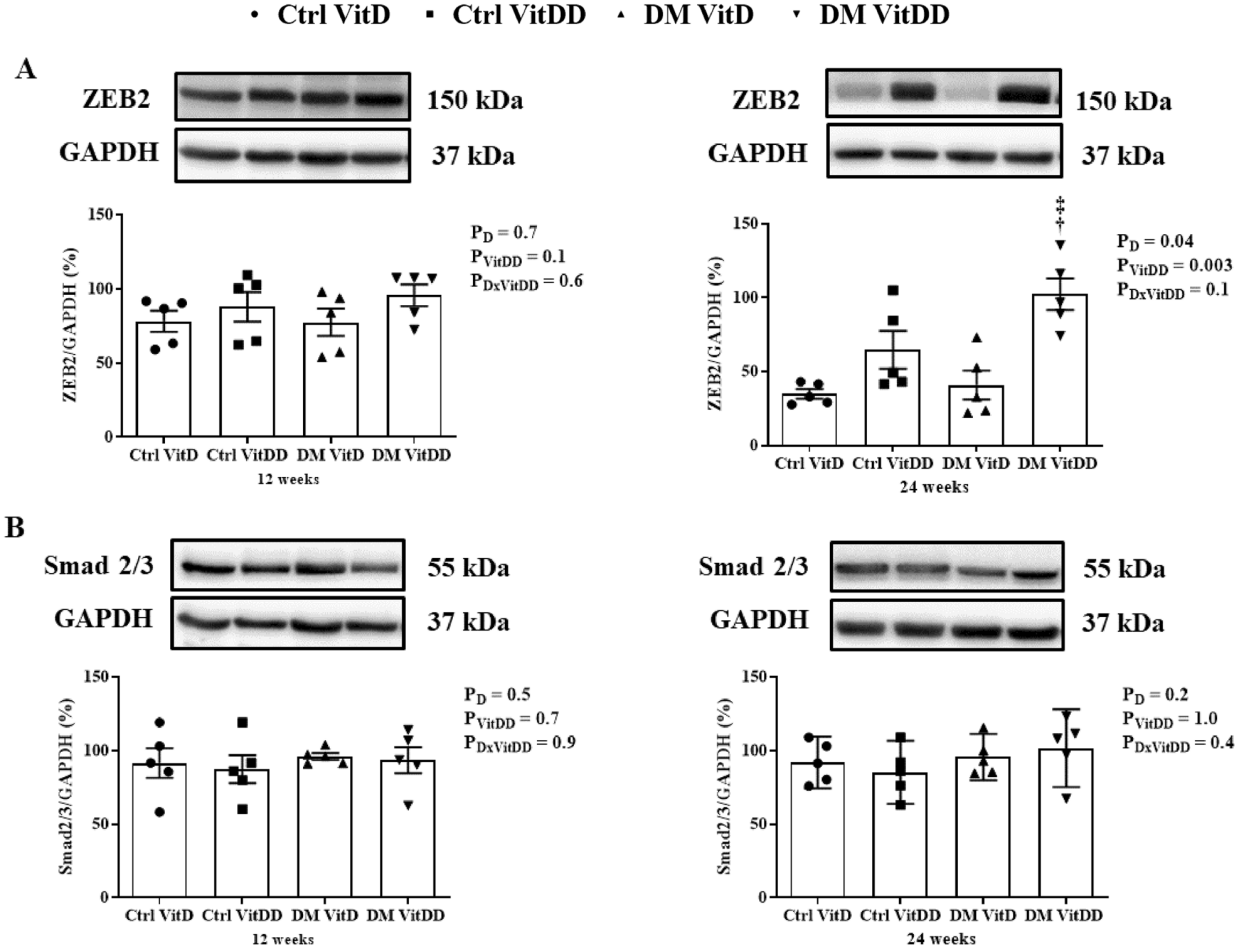
Protein expression of the epithelial-mesenchymal transition markers in renal tissue of control (Ctrl) and diabetic (DM) rats vitamin D deficient (VitDD) or not (VitD) at 12 and 24 weeks after diabetes induction. **A** ZEB2 and **B** Smad2/3 protein levels. ^†^versus Ctrl VitDD, ^‡^versus DM VitD. PD: interaction between DM VitD and Ctrl VitD and DM VitDD and Ctrl VitDD; PVitDD: interaction between Ctrl VitDD and Ctrl VitD and DM VitDD and DM VitD; PDxVitDD: interaction between DM VitDD and DM VitD.

The DM VitDD group presented an increase of ZEB2 protein expression at 24 weeks after diabetes induction compared to Ctrl VitDD and DM VitD groups (*P* = 0.003) (Fig. 8A). No statistical difference for Smad2/3 between the experimental groups was observed (*P* > 0.05) (Fig. 8B).

### TGF-β1 levels

**Fig. 9:**
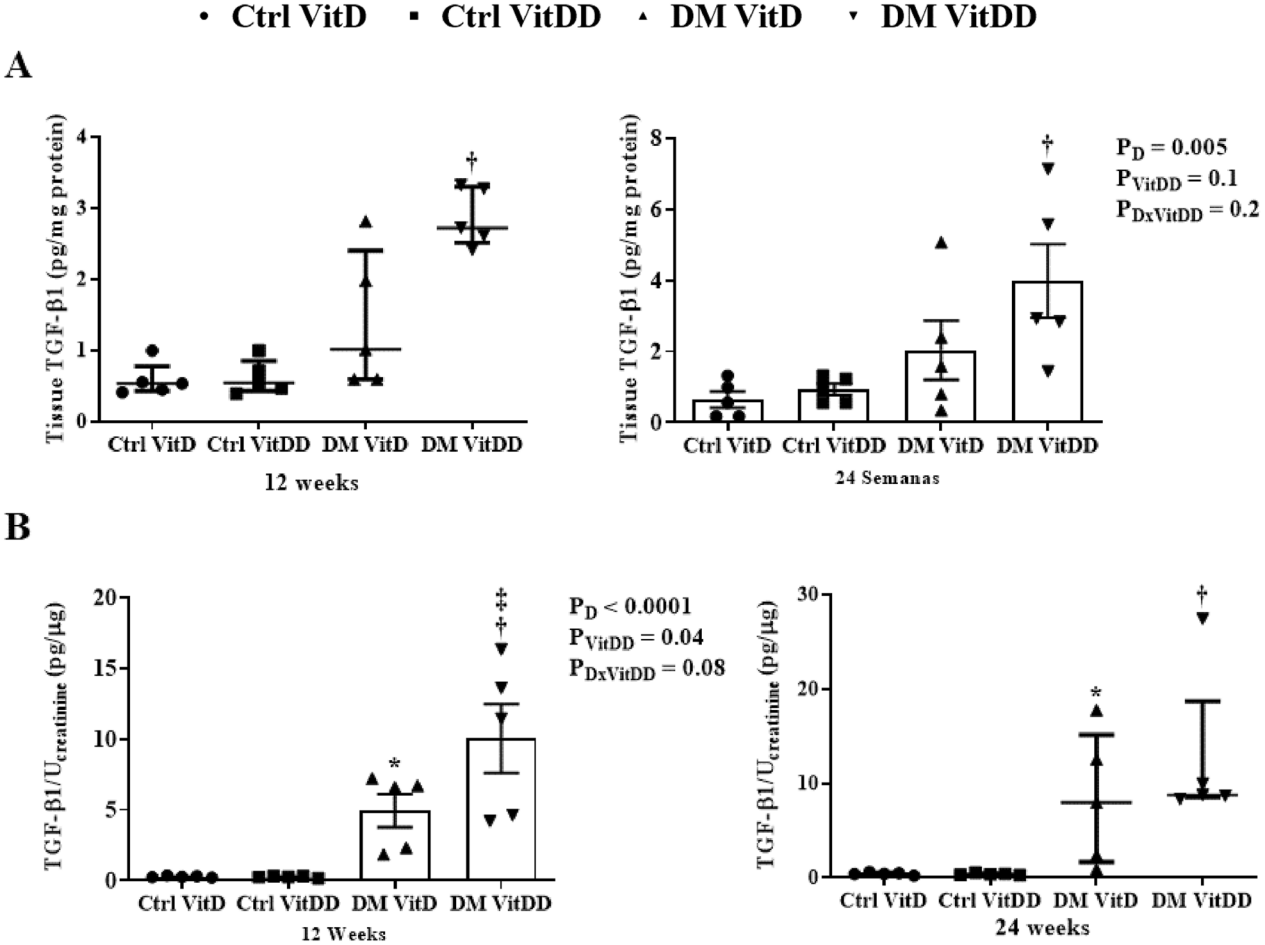
TGF-β1 levels in renal tissue and urine of control (Ctrl) and diabetic (DM) rats vitamin D deficient (VitDD) or not (VitD) at 12 and 24 weeks after diabetes induction. **A** Renal tissue levels and **B** urinary excretion of TGF-β1. *versus Ctrl VitD, ^†^versus Ctrl VitDD, ^‡^versus DM VitD. PD: interaction between DM VitD and Ctrl VitD and DM VitDD and Ctrl VitDD; PVitDD: interaction between Ctrl VitDD and Ctrl VitD and DM VitDD and DM VitD; PDxVitDD: interaction between DM VitDD and DM VitD.

The tissue levels and urinary excretion TGF-β1 were higher in the rats of the DM VitD and DM VitDD groups when compared to Ctrl VitD and Ctrl VitDD groups (*P* < 0.05) (Fig. 9A, B). However, this increase was more intense in the DM VitDD group (Fig. 9A, B) and significantly different in urinary excretion at 12 weeks compared to the DM VitD group (*P* = 0.04) (Fig. 9B).

## Discussion

This study evaluated the VitD deficiency influence in the progression of DKD and the ZEB1/ZEB2 contribution in this process. Our results demonstrated that VitD-deficient diabetic rats have an increase in the renal structural changes, in the transdifferentiating markers in the different kidney cells, ZEB1/2 expressions, TGF-β1 levels, a decrease in renal function and miR-200b downregulation compared to the diabetic rats with normal VitD levels.

Our data demonstrated an increase in blood pressure (BP) in VitD-deficient diabetic rats eight weeks after diabetes induction. Clinical and experimental studies have reported an increased prevalence of hypertension associated with VitD deficiency [29, 30]. In a previous study by our laboratory, it was demonstrated that the increase in BP of the adult offspring of mothers submitted to VitD deficiency during pregnancy and the period of renal development was related to the increase in the renin renal tissue expression and of the receptor for type 1 angiotensin II [28]. Andersen et al. (2015) demonstrated that VitD depletion led to increasing endogenous and human renal expression of renin mRNA in dTGR rats (double-transgenic rats expressing human renin and angiotensinogen) and increased hypertension in this experimental model [29].

In the 24^th^ week after the diabetes induction, we observe a reduction in VDR expression in the VitD-deficient rats. As in our findings, Bragança et al. (2018) also showed reduced VDR expression in VitD-deficient 5/6 nephrectomized and sham rats [31]. Studies demonstrated that VDR-knockout mice increase the glomerulosclerosis and tubulointerstitial fibrosis in the UUO model [32], deteriorate renal function and tubular injury in cisplatin-induced acute kidney injury (AKI) [33] and contribute to severe albuminuria, worsen of tubular injuries, an increase of inflammation and to defective autophagy in the DKD [34].

Hypovitaminosis D is frequently related to the development of mild albuminuria [13], sustained decline in the renal function and increase glomerular and tubular lesions in diabetic patients [35–37]. In our study, VitD deficiency worsens changes in renal structure and function in diabetic rats. Previous reports demonstrated that VitD deficiency increases glomerulosclerosis and relative interstitial area, and impairs renal function in the progression to CKD after ischemia/reperfusion (I/R) and in the 5/6 nephrectomized rats [31, 38]. Studies suggest that these alterations occur due to a lower VDR modulation [39], local overactivation of the renin angiotensin aldosterone system [28, 32, 40] and/or secondary factors such as elevated PTH levels in VitD deficiency [41]. However, the mechanisms by which VitD deficiency contributes to the worsen of these changes, mainly in DKD, should be further explored.

The EMT is an important mediator in the development and progression of DKD [4, 5] During this fibrotic process, the renal cells change their phenotype to myofibroblasts and start to express mesenchymal marker-positive epithelial cells such as desmin, vimentin and α-SMA [42]. Our data demonstrated an increase in EMT in the VitD-deficient diabetic rats. Similar to our findings, previous studies have also demonstrated that VitD deficiency is related to increase expression of vimentin and α-SMA in tubular and tubulointerstitial cells, respectively, of the rats submitted to a high-fat diet [43] and in rats euthanized sixty days after I/R renal injury [38]. An increase in the desmin, vimentin and α-SMA expressions may contribute to the accumulation of ECM and consequent glomerulosclerosis and tubulointerstitial fibrosis considered the end common pathway to all progressive kidney diseases and which can culminate in ESRD [42, 44]. Despite this, the mechanisms that lead to the accumulation of these proteins in a DKD model in VitD deficiency are still poorly studied. In our study, we investigated the ZEB1/2 participation, an important transcriptional co-repressors related to the EMT process. Our data demonstrated an increase in the expression of these transcription factors. Associated with this, was observed an increase in TGF-β1 levels and miR-200b downregulation in VitD-deficient diabetic rats, main regulators of ZEB1/2 expression.

The relevance of both ZEB proteins is mainly due to their roles in tumorigenesis related to cell proliferation and tumor invasion, metastasis and resistance to chemotherapy drugs [45]. ZEB1/2 bind directly to the promoter of genes encoding epithelial proteins, suppressing their expression while inducing the expression of proteins with mesenchymal characteristics [7]. Several studies have also demonstrated the importance of the ZEB1/2 signaling pathway in renal fibrosis [6, 8, 9, 46]. Wei et al. (2014) demonstrated that aldose reductase negatively regulates the miR-200a expression and this was associated with the upregulation of TGF1/2 and ZEB1/2 expressions and consequent increase in fibrogenesis and EMT in mesangial cell culture and diabetic mice [46]. Studies in renal tubular cells culture demonstrated that overexpression of all members of miR-200 family leds to the the reduction of ZEB1/2 and TGF-β1 gene and protein expression at the early stage of EMT [8], and miRNA-200b alone was able to suppress EMT induced by TGF-β1 via reducing ZEB1/2 expression [47]. Additionally, in vitro studies demonstrated that miR-200b overexpression decreases the proliferation, migration and metastasis in lung [48] and breast [49] cancer cells. Taken together, these studies show that regulation of ZEB1/2 by the miR-200 family and TGF-β plays an essential role in renal EMT. To date, no studies were found showing the relationship between VitD deficiency and ZEB1/2 regulation in renal diseases. Nevertheless, it was demonstrated that VitD deficiency contributed to increasing expression of TGF-β1 and ZEB1, which in turn, mediated EMT in bleomycin-induced pulmonary fibrosis [50].

In conclusion, our data demonstrate that VitD deficiency provided a favorable condition for ZEB1/2 upregulation and miR-200b downregulation, which contributed to the EMT process and consequently increase in the structure and function renal changes in diabetic rats.

## Supplementary information

Supplementary Information

## Data Availability

The author confirms that all data generated or analyzed during this study are included in this published article.
