# Supplementary material for: Vitamin D deficiency contributes to the diabetic kidney disease progression via increase ZEB1/ZEB2 expressions
Source: Nutr Diabetes. 2023 Jul 1;13:9. doi: 10.1038/s41387-023-00238-2 (PMC10313688; doi:10.1038/s41387-023-00238-2)
Supplement: Supplementary file 1 — Supplementary Information [file 41387_2023_238_MOESM1_ESM.pdf]

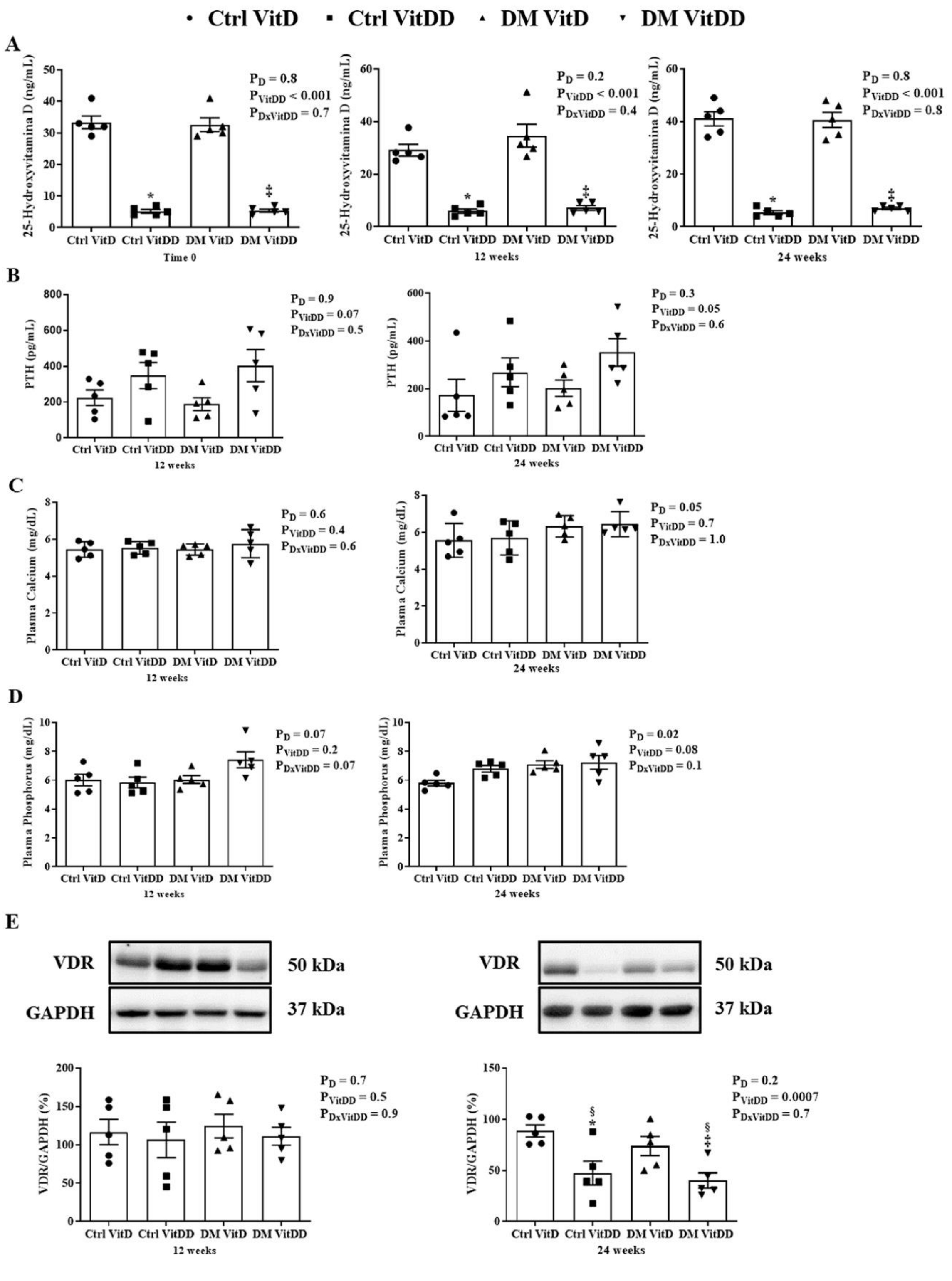

Supplementary Figure 1. Experimental model characterization. **A**, Serum Levels of 25-Hydroxyvitamin D; **B**, PTH, parathyroid hormone; **C**, Plasma Calcium; **D**, Plasma Phosphorus; **E**, protein levels of vitamin D receptor (VDR) of control (Ctrl) and diabetic (DM) rats deficient in vitamin D (VitDD) or not (VitD). *versus Ctrl VitD, †versus Ctrl VitDD, ‡versus DM VitD, §versus DM VitDD 12 weeks after diabetes induction. P_D: interaction between DM VitD and Ctrl VitD and DM VitDD and Ctrl VitDD; P_{VitDD}: interaction between Ctrl VitDD and Ctrl VitD and DM VitDD and DM VitD; $P_{DxVitDD}$: interaction between DM VitDD and DM VitD.

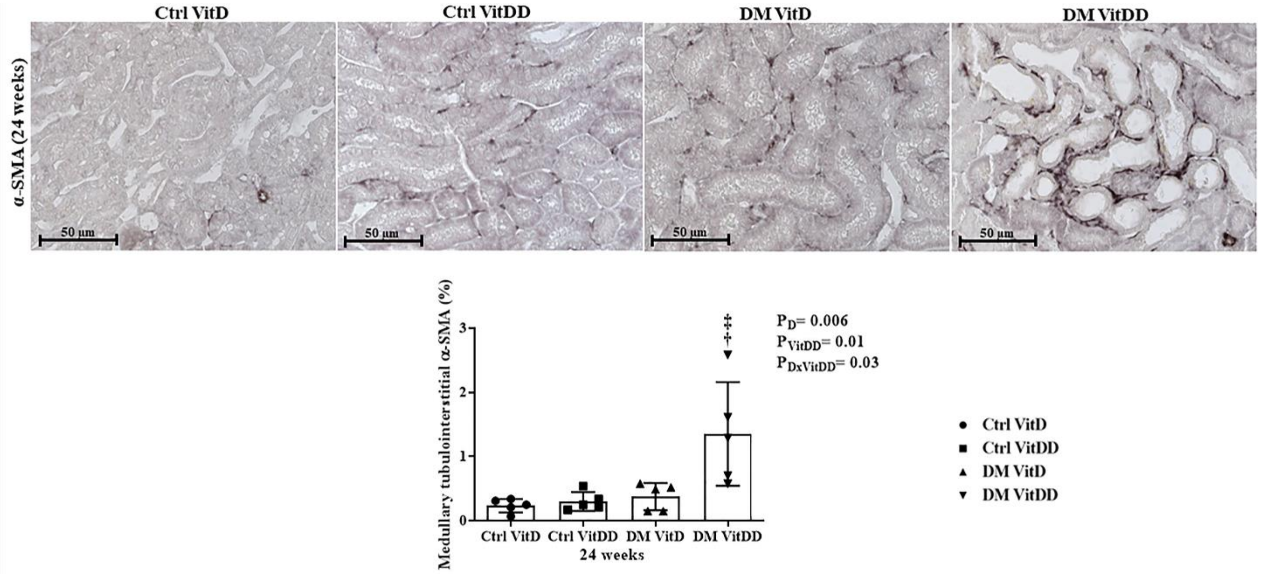

Supplementary Figure 2. Immunolocalization and quantification for α-SMA in the outer medulla of controls (Ctrl) and diabetic (DM) rats vitamin D deficient (VitDD) or not (VitD) at 12 and 24 weeks after diabetes induction. [†]versus Ctrl VitDD, [‡]versus DM VitD. P_D: interaction between DM VitD and Ctrl VitD and DM VitDD and Ctrl VitDD; P_{VitDD}: interaction between Ctrl VitDD and Ctrl VitD and DM VitDD and DM VitD; $P_{DxVitDD}$: interaction between DM VitDD and DM VitD. Original magnification x400.

Supplementary Table 1. Metabolic and renal function data of controls (Ctrl) and diabetic (DM) rats vitamin D deficients (VitDD) or not (VitD).

Variable	GROUP				ANOVA		
	Ctrl VitD	Ctrl VitDD	DM VitD	DM VitDD	P_D	P_{VitDD}	$P_{DxVitDD}$
**FBG (mg/dL)**							
0	79 ± 4	79 ± 5	89 ± 3	84 ± 3	0.1	0.2	0.1
4	64 ± 2	64 ± 3	348 ± 10*	316 ± 13†	< 0.0001	0.7	0.6
8	87 ± 7	92 ± 7	318 ± 15*	320 ± 17†	< 0.0001	0.6	0.8
12	90 ± 5	99 ± 5	357 ± 7*	363 ± 15†	< 0.0001	0.1	0.7
16	94 ± 4	98 ± 8	376 ± 20*	383 ± 8†	< 0.0001	0.3	0.8
20	92 ± 6	91 ± 6	361 ± 16*	347 ± 11†	< 0.0001	0.1	0.2
24	84 ± 2	87 ± 2	362 ± 9*	363 ± 9†	< 0.0001	0.7	0.3
**MAP (mmHg)**							
0	81 ± 8	89 ± 3	86 ± 5	84 ± 2	1.0	0.3	0.1
4	94 ± 4	92 ± 3	96 ± 3	105 ± 5	0.7	0.4	0.2
8	94 ± 3	96 ± 3	97 ± 4	110 ± 5†‡	0.04	0.05	0.2
12	96 ± 4	94 ± 3	109 ± 2*	107 ± 5†	0.003	0.6	0.9
16	91 ± 4	84 ± 3	88 ± 6	93 ± 3†	0.4	0.8	0.1
20	90 ± 2	92 ± 2	83 ± 3	90 ± 6	0.2	0.2	0.6
24	88 ± 2	90 ± 3	86 ± 4	92 ± 3	0.7	0.3	0.5
**UAE (mg/24h)**							
0	0.03 ± 0.006	0.03 ± 0.008	0.03 ± 0.008	0.03 ± 0.009	1.0	0.9	0.7
4	0.06 ± 0.08	0.07 ± 0.09	1.1 ± 0.3*	1.3 ± 0.4†	0.0004	0.6	0.6
8	0.1 ± 0.02	0.1 ± 0.02	1.2 ± 0.3*	1.6 ± 0.2†	< 0.0001	0.4	0.4
12	0.1 ± 0.005	0.1 ± 0.005	1.6 ± 0.5*	1.9 ± 6.0†	0.0007	0.8	0.8
16	0.1 ± 18	0.1 ± 0.02	1.7 ± 0.3*	2.4 ± 7.9†	0.0003	0.4	0.4
20	0.1 ± 3	0.1 ± 0.003	3.6 ± 1.7*	6.8 ± 4.5†	< 0.0001	0.09	0.09
24	0.3 ± 0.1	0.3 ± 0.02	4.0 ± 1.6*	8.7 ± 4.2†	0.02	0.3	0.3

Supplementary Table 1. Data are presented as mean±SEM and in median (25th and 75th percentile), n=5/each. FBG, Fasting Blood Glucose; MAP, Mean Arterial Pressure; UAE, Urinary Albumin Excretion; Time in weeks (0, 4, 8, 12, 16, 20, 24). *versus Ctrl VitD; †versus Ctrl VitDD; ‡versus DM VitD. PD: interaction between DM VitD and Ctrl VitD and DM VitDD and Ctrl VitDD; PVitDD: interaction between Ctrl VitDD and Ctrl VitD and DM VitDD and DM VitD; PDxVitDD: interaction between DM VitDD and DM VitD.
